# Circular RNA CircEZH2 Suppresses Transmissible Gastroenteritis Coronavirus-induced Opening of Mitochondrial Permeability Transition Pore via Targeting MiR-22 in IPEC-J2

**DOI:** 10.7150/ijbs.36532

**Published:** 2019-07-25

**Authors:** Xiaomin Zhao, Xuelian Ma, Jianxiong Guo, Mi Mi, Kaili Wang, Chuyi Zhang, Xiaoyi Tang, Lingling Chang, Yong Huang, Dewen Tong

**Affiliations:** College of Veterinary Medicine, Northwest A&F University, Yangling, Shaanxi 712100, P.R. China

**Keywords:** transmissible gastroenteritis coronavirus virus, circular RNA, microRNA, mitochondrial permeability transition pore, NF-κB

## Abstract

Transmissible gastroenteritis (TGE) is a contagious and infectious disease that is characterized by severe vomiting and diarrhea of swine , especially piglet, and caused by transmissible gastroenteritis coronavirus (TGEV) . TGEV infection provokes mitochondrial damage of porcine intestinal epthelial cell (IPEC), which is responsible for inflammation and cell death. In our previous study, we have demonstrated that circular RNA circEZH2 was down-regulated during TGEV infection and promoted the activation of nuclear factor kappa-light-chain-enhancer of activated B cells (NF-κB) via targeting miR-22 in porcine intestinal epithelial cell line (IPEC-J2). Activation of NF-κB is an important factor for mitochondrial damage. Mitochondrial permeability transition pore (mPTP) opening is a key reason for mitochondrial damage. So, we speculate that circEZH2 may regulate TGEV-induced mPTP opening via NF-kB pathway. In the present study, we found that mPTP opening of IPEC-J2 was occured during TGEV infection and suppressed by circEZH2 via attaching miR-22. Hexokinase 2 (HK2) and interleukin 6 (IL-6) were identified as the targets of miR-22. Silencing HK2 enhanced TGEV-induced mPTP opening, while no effect on NF-κB pathway. Silencing IL-6 promoted TGEV-induced mPTP opening and inhibited NF-κB pathway. Inhibitor of NF-κB increased TGEV-induced mPTP opening. The data revealed that TGEV-induced mPTP opening was regulated via two pathways: circEZH2/miR-22/HK2 axis and circEZH2/miR-22/IL-6/NF-κB axis.

## Introduction

Coronaviruses (CoVs) are the causative agents of human and a variety of animal diseases. In human, CoVs evoke respiratory tract infection and cause common cold or acute respiratory distress syndrome (ARDS) 1-3. In animals, CoVs also lead to life-threatening diseases, such as severe enteric and respiratory tract infections, and are economically important pathogens^4^-^7^. TGEV is a member of *Coronaviridae* and causes TGE. TGE is a highly contagious gastroenteric disease of swine and results in high mortality of less than 2-week-old piglet. TGEV infection can evoke mitochondrial damage, inflammation, and cell death^8^, ^9^. TGEV-induced mitochondrial damage is a key factor of inflammation and is caused by abnormal opening of mitochondrial permeability transmission pore (mPTP)^10^. Persistent opening of mPTP can lead to decrease of the mitochondrial membrane potential (△ψm), reduction of ATP production, release of ROS from mitochondria, finally mitochondrial damage^11^-^14^. Viral infection is one of the important factors for mitochondrial injury^15^-^18^. Studies have confirmed that TGEV infection can cause intracellular mitochondrial injury. For example, TGEV infection of IPEC-J2 can cause intracellular mitochondrial autophagy^19^. PK-15 cells infected with TGEV can appear mitochondrial injury, showing an increase of mitochondrial Ca^2+^ and mitochondrial membrane permeability^20^, ^21^.

miR-22 has a strong correlation with NF-κB pathway and mitochondrial damage. Recently, it was reported that miR-22 inhibited PI3K/Akt/NF-κB signaling in tongue squamous cell carcinoma (TSCC) cells via targeting KAT6B^22^. miR-22 prevents activation of interferon regulating factor 3 (IRF3) and NF-κB pathway^23^. In H9c2 cardiomyocyte, it was found that miR-22 aggravated H/R-induced mitochondrial damage^24^.

Viral infection can regulate NF-κB and mPTP opening. Hepatitis B virus x protein (HBx) inhibits HBV-induced apoptosis through activating NF-κB in primary rat hepatocytes. Suppression of mPTP results in HBx-induced apoptosis, indicating NF-κB is strongly linked to mPTP^25^. Activation of NF-κB by TNF-α decreases calcium induced mPTP opening via repressing ANT1^26^. Recently, we reported that mitochondrial damage was caused by TGEV infection. Moreover, circEZH2 enhanced activation of NF-κB pathway via sponging miR-22 during TGEV-induced inflammation process 21. circEZH2 is comprised of exon 2 to 8 of EZH2 (ENSSSCG00000028840) and is derived from 109, 411, 982 bp -109, 431, 724 bp region of the sense strand of Chromosome 9. NF-κB pathway is an important pathway that related to regulating mitochondrial damage and inflammation^27^. So, we propose a hypothesis that circEZH2 may regulate mPTP opening via miR-22.

To prove the hypothesis, we investigated the effects of circEZH2 and miR-22 on TGEV-induced mPTP opening and NF-κB pathway, identified the targets of miR-22, and tested the effects of miR-22 targets on mPTP opening and NF-κB pathway. The data demonstrated that circEZH2 attenuated TGEV-induced mPTP opening via attaching miR-22. miR-22 promoted mPTP opening by targeting HK2 and IL-6. Moreover, IL-6 inhibited mPTP opening by activating NF-κB pathway, while HK2 had no significant impact on activation of NF-κB pathway. The data will provide a potential pharmacologic target for prevention and control of TGE.

## Materials and Methods

### Antibodies, cells, and virus

β-actin monoclonal antibody was purchased from Santa Cruz (US). Horseradish peroxidase (HRP)-conjugated secondary antibody was purchased from Pierce (US). Hexokinase Ⅱ (HK2) and Phospho-NF-κB p65 (p-p65) Rabbit monoclonal antibody was purchased from Cell Signaling Technology (US). Dylight594-conjugated secondary antibody was purchased from Genshare Biological (CHN). IPEC-J2 cell line was gifted from Zhanyong Wei (Henan Agricultural University, CHN). Cells were cultured in Dulbecco's Modified Eagle Medium (DMEM)/F-12/HAM (Thermo Fisher Scientific, US), which was supplemented with 100 IU of penicillin and 100 mg of streptomycin per ml, at 37℃ in an incubator with 5% CO_2_. The TGEV Shaanxi strain was separated from TGEV-infected piglets.

### Animal experiments

Six new-born piglets from the same sow, which are free of TGEV, porcine epidemic diarrhea virus (PEDV), Porcine Rotavirus (PoRV), and porcine circovirus type 2 (PCV2), were randomly assigned to 2 groups, 3 piglets per group, and housed separately. Group 1 was orally infected with 5 ml phosphate-buffered saline (PBS), named Mock group as the uninfected control. Group 2 piglets were orally infected with 5 ml of 10^7^ median (50%) tissue culture infective doses (TCID50)/ml of TGEV as experimental group. All piglets were euthanized at 48 h after diarrhea and the jejunum tissues were immediately sampled.

### Histological analysis

Jejunum tissue were fixed in 4% paraformaldehyde for 48 h at room temperature, embedded in paraffin, and sliced. The morphological observation of porcine jejunum was conducted after hematoxylin-eosin (HE) staining using Aperio Digital Pathology Slide Scanners (Leica, GER).

### Transmission electron microscopy (TEM)

The jejunum tissue samples were cut into smaller pieces (1 mm × 1 mm × 3 mm). The monolayer of IPEC-J2 cells infected with TGEV or Mock were fixed in situ for 24 h with a mixture of 2.5% glutaraldehyde and 2% formaldehyde in PBS (pH 7.2) for 6 h at room temperature. The samples were removed from the dishes and transferred to Eppendorf tubes. After centrifugation in a microcentrifuge at 300 g for 5 min, the samples were washed with PBS and postfixed with a mixture of 1% osmic acid in distilled water for 4 h at 4℃. After washing four times with PBS, samples were dehydrated with graded ethanol (30, 50, 70, 90, and 100%) for 10 min each concentration at room temperature. The tissues were sequentially infiltrated in LR White Resin (Sigma-Aldrich, US) and ethanol (1:3) for 2 h at room temperature, in the LR White Resin and ethanol (1:1) for 8 h at room temperature, in LR White Resin and ethanol (3:1) for 24 h at room temperature, and in LR White Resin for 2 days at room temperature. Ultrathin (75-nm-thick) sections of the samples were stained with both uranyl acetate and lead citrate by standard procedures. All sections were observed and photographed with a FEI Tecnai G2 Spirit Bio-TWIN transmission electron microscope (FEI, US).

### Mitochondrial Permeability Transition Pore (mPTP) activity

Cells were infected with TGEV at 1.0 MOI for 24 h. Meanwhile, the mock infection was carried out. Each sample was treated with 5 μl of BBcellProbe^TM^ M61 probe (200 μM) (BestBio, Shanghai, CHN) for 5 min and quencher for 15 min at 37°C. The fluorescence was measured by flow cytometry, with 15, 000 events collecting. Opening of mPTP results in a decrease of fluorescence.

### Fluorescence in situ hybridization (FISH)

Specific probes to circEZH2 sequence and miR-22 were synthesized by Genepharma (Shanghai, CHN) and used in in situ hybridization (The sequences are shown in Table S3). In brief, CY3-labelled probe was specific to circEZH2. FAM-labelled probe was specific to miR-22. Nuclei were counterstained with 4,6-diamidino-2-phenylindole (DAPI). All the procedures were conducted according to the manufactory's instruction (Genepharma, Shanghai, CHN). Specimens were analyzed using LEICA TCS SP8 confocal microscopy (Leica, GER).

### RNA extraction

The IPEC-J2 cells were infected with TGEV (MOI=1.0) when 70% confluent in the culture dish and harvested at 24 h post infection (hpi). The villus of jejunum was scraped and immediately frozen in liquid nitrogen for RNA extraction. RNA was extracted using Trizol reagent (Invitrogen, US) according to the manufacturer's instructions. RNA concentration and quality were measured by NanoDrop1000 spectrophotometer (NanoDrop Technologies, US).

### Quantification of miRNAs, circRNAs, and mRNAs by qRT-PCR

The total RNA was reversely transcribed by M-MLV reverse transcriptase (Invitrogen, US). Especially, for reverse transcription of circRNAs, the total RNA was treated with RNase R (Epicentre, US) to remove liner RNA and then was subjected to reverse transcription. qRT-PCR was performed as described previously on iQ5 real-time PCR System (Bio-Rad, US) 28. The relative quantification of miRNAs was normalized to U6 using 2^-ΔΔCt^ method. The relative quantification of mRNAs and circRNAs were normalized to β-actin using the 2^-ΔΔCt^ method (The primers are shown in Table S4).

### Prediction and analysis of miR-22 targets

RNAhybrid (v2.1.2) + svm_light (v6.01), Miranda (v3.3a) and TargetScan (V7.0) were used to predict the target genes of miR-22. A intersection between miR-22 targets and identified genes by sequencing was obtained and presented by Venn diagrams. GO term analysis of the target genes were performed by DAVID Bioinformatics Resources 6.8 (https://david.ncifcrf.gov/home.jsp). The miR-22-mRNA regulatory network was constructed by Cytoscape (v3.5.1) (http://www.cytoscape.org/).

### Luciferase reporter assay

3' UTRs of candidate target genes containing the binding site of miR-22 were respectively amplified by PCR and were cloned into the vector psiCHECK-2 (Promega, US). To obtain mutation of miR-22 complementary sites within the 3' UTR of HK2 and IL-6, the binding sites of miR-22 seed region in 3' UTRs were mutated following a mutagenesis protocol 29 (The primers are shown in Table S4). The miR-22 mimics, miR-22 mimics control, miR-22 inhibitor, and miR-22 inhibitor control were designed and synthesized by Genepharma (Shanghai, CHN) (The sequences are shown in Table S3). For the luciferase reporter assay, IPEC-J2 cells were seeded in 24-well plate and then co-transfected with 100 ng plasmid and 100 nM of miR-22 mimics, or miR-22 inhibitor, or control, using Lipofectamine 3000 (Invitrogen, US) according to the manufacturer's instructions. At 48 h post transfection (hpt), the luciferase activities were measured using Dual-Glo Luciferase Assay System (Promega, US) following the manufacturer's manual.

### Analysis of Protein-Protein interactions

HK2 was searched against STRING database (version 10.0) for protein-protein interactions (https://string-db.org/cgi/input.pl).

### Construction of recombinant plasmid for circEZH2 overexpression

The full-length cDNA of circEZH2 was synthesized by Genebio (Shanghai, CHN) and cloned into the pcircRNA between two frames. The recombinant plasmid was named pcircRNA-circEZH2.

### Transfection of siRNAs and plasmids

IPEC-J2 cells were maintained in a 6-well plate with DF-12 medium supplemented with 10% FBS and not collected until 50-70% confluent. Cells were transfected with siRNAs (Shanghai, CHN) (The sequences are shown in Table S3), or plasmids using Lipofectamine 3000 (Invitrogen, US) according to the manufacturer's instructions. Countermeasure and overexpression effect was examined by RT-qPCR using RNA extracted 48 hours after transfection.

### Western blot analysis

Cells were treated with RIPA lysis buffer containing phenylmethyl sulonylfluoride (PMSF). Protein concentration was determined using BCA Protein Assay Regent (Pierce, US). Proteins were separated using sodium dodecyl sulfate-polyacrylamide gel elecrophoresis (SDS-PAGE) and subsequently transferred onto polyvinylidene difluoride (PVDF) membrane (Millipore Corp, US). The PVDF membrane was blocked with 5% non-fat dry milk for 2 h at room temperature and then incubated with primary antibodies overnight at 4 ℃. Subsequently, the membrane was incubated with HRP-conjugated secondary antibody at room temperature for 1 h. The signal was detected with enhanced chemiluminescence (ECL) kit (Genshare, CHN).

### Statistics

The data was presented as the means ± SEM. Statistical significance was analyzed by unpaired Student's t-test, where * *p* < 0.05, ** *p* < 0.01.

## Results

### TGEV induced opening of mPTP

**Figure 1:**
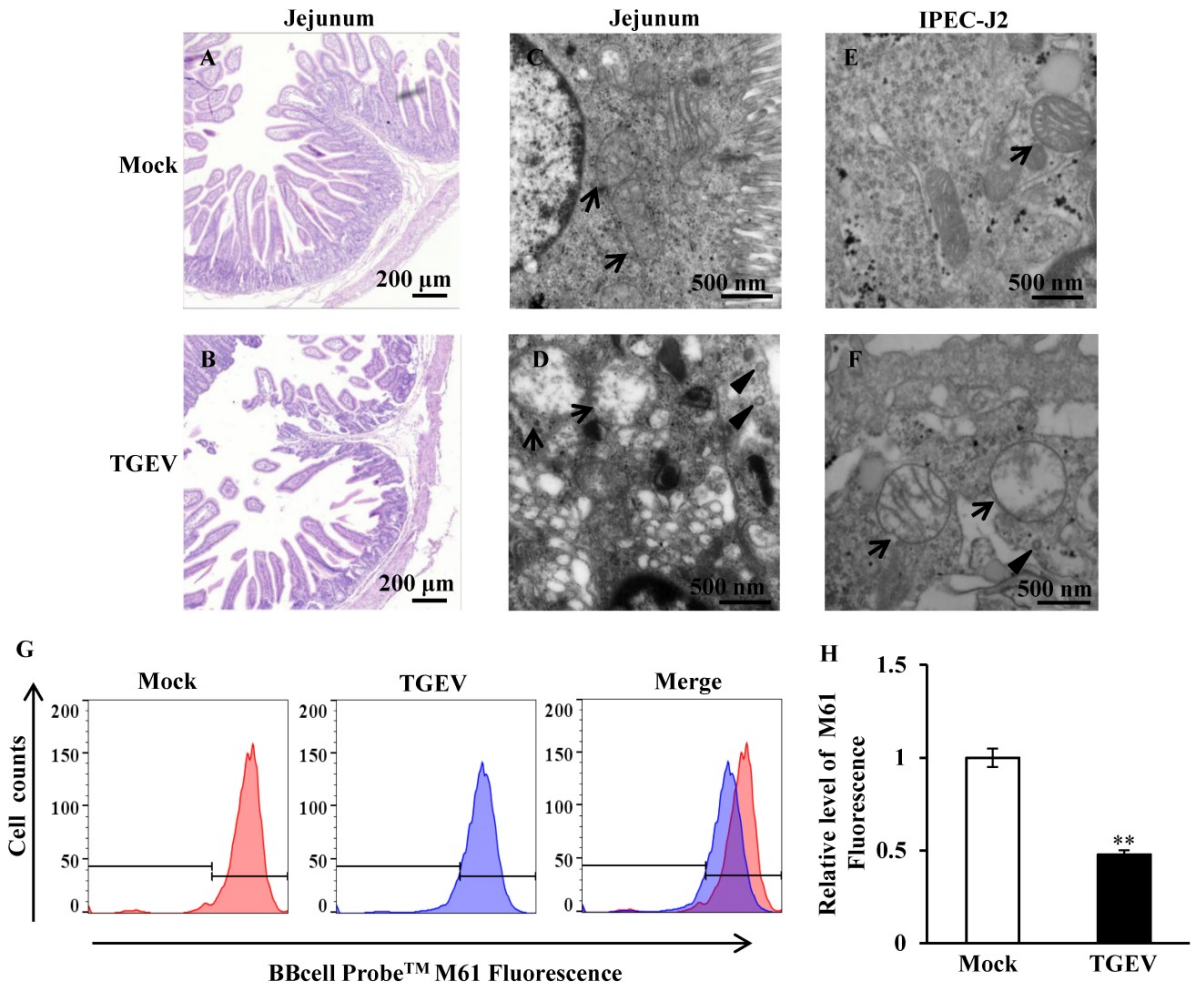
**TGEV infection resulted in mitochondrial damage and mPTP opening.** (A) and (B) Histological changes of piglet jejunum infected with TGEV. (C) and (D) Ultrastructure changes of mitochondria in jejunum IPEC of piglet in response to TGEV infection. (E) and (F) Ultrastructure changes of mitochondria in IPEC-J2 in response to TGEV infection. Black arrows indicate mitochondrion. Black triangles indicate viral particle. (G) and (H) The degree of mPTP opening of IPEC-J2 infected with TGEV. The fluorescence was measured via FCM and quantified with Fluorescence Activated Cell Sorting (FACS). ** *p* < 0.01.

The jejunum of the piglets were fixed, embedded, sliced, and stained using HE. The sections were observed using light microscope. The results showed that the jejunum villi of mock-infected piglets were long and slender (Figure 1A), while the villi of TGEV-infected piglets were shortened and fell away (Figure 1B). Ultrastructural changes were observed using transmission electron microscopy. The results showed virus particles appeared in the cytoplasmic matrix and mitochondria swelling was found in TGEV infected group (Figure 1C to F). The fluorogenic probe, BBcell ProbeTM M61, was used to evaluate the effect of TGEV on mPTP opening in IPEC-J2. These results indicated that the fluorescent value of TGEV-infected cells showed a significant decrease in comparison with the mock-infected cells (Figure 1G and H).

### circEZH2 suppressed mPTP opening induced by TGEV infection

**Figure 2:**
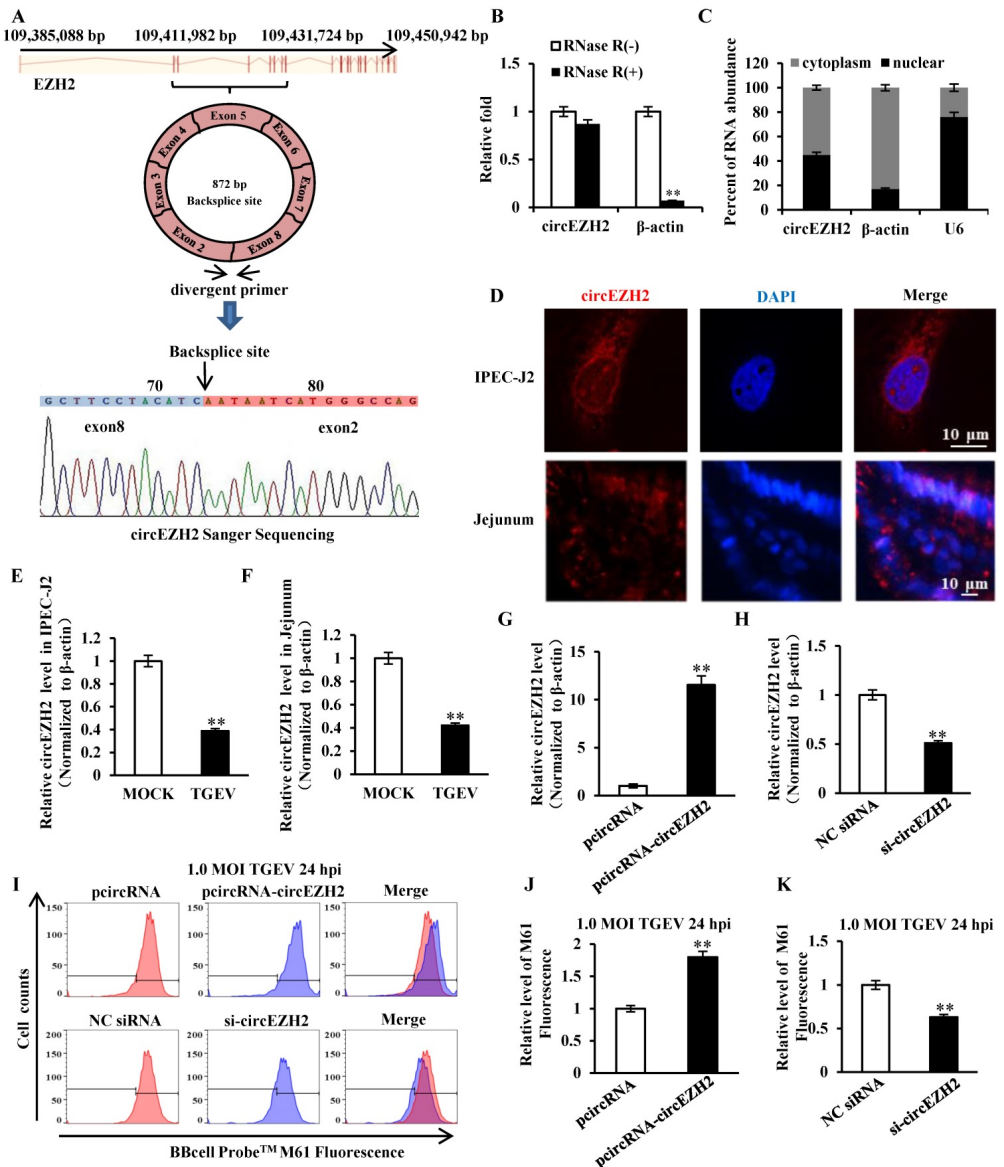
**circEZH2 localized at both nucleus and cytoplasm, and suppressed TGEV-induced mPTP opening.** (A) The genomic loci of circEZH2 and the Sanger sequence of backsplice site of circEZH2. Arrows represent divergent primers binding to the genome region of circEZH2. (B) qRT-PCR analysis of β-actin and circEZH2 after Rnase R treatment. (C) qRT-PCR analysis of circEZH2 in cytoplasm and nuclear. mRNA level ofβ-actin in cytoplasm and U6 level in nuclear fraction were respectively referred as quality controls of cytoplasm and nuclear fractions. (D) circEZH2 was localized at both nucleus and cytoplasm in IPEC-J2 and jejunum IPEC of piglet. (E) Fold change of circEZH2 level in IPEC-J2 in response to TGEV infection. (F) Fold change of circEZH2 level in jejunum IPEC of piglet in response to TGEV infection. (G) Overexpression effect of pcircRNA-circEZH2. (H) Silencing effect of si-circEZH2. (I) The effect of circEZH2 on TGEV-induced mPTP opening in IPEC-J2. (J) and (K) Quantification of mPTP opening level of Figure 2I. ** *p* < 0.01.

Our previous circRNAs sequencing date (available in the Sequence Read Archive with accession number SRP128150) demonstrated that some circRNAs differentially expressed during TGEV infection in IPEC-J2. The circular RNA ssc_circ_009380 was differentially down-regulated during TGEV infection^21^. Analysis of ssc_circ_009380 sequence showed that it was comprised of exon 2 to 8 of EZH2 (ENSSSCG00000028840) that derived from 109, 411, 982-109, 431, 724 bp regions of the sense strand of Chromosome 9 (Figure 2A). Therefore, circular RNA ssc_circ_009380 was named "circEZH2". To confirm the circular characteristics of circEZH2, we next digested total RNA with or without RNase R. Compared to the linear β-actin mRNAs, circEZH2 was obviously resistant to Rnase R (Figure 2B). In addition, we found circEZH2 was localized at both nuclear and cytoplasm (Figure 2C and D). Meanwhile, The levels of circEZH2 were measured by qRT-PCR and FISH. The results revealed that circEZH2 was down-regulated during TGEV infection *in vitro* (Figure 2E and Figure S1) and *in vivo* (Figure 2F and Figure S1), which is correlated with the Solexa high-throughput sequencing. To investigate the impact of circEZH2 on mPTP opening induced by TGEV, IPEC-J2 cells were transfected with pcircRNA-circEZH2 (or pcircRNA, si-circEZH2, NC siRNA) and subsequently infected with TGEV at 1 MOI for 24 h. The circEZH2 level was significantly increased by pcircRNA-circEZH2 and remarkably decreased by si-circEZH2 (Figure 2G and H). The level of mPTP opening was tested using BBcellProbeTM M61 probe. These results suggested fluorescence intensity of cells infected with TGEV significantly increased by pcircRNA-circEZH2 and significantly decreased by si-circEZH2 in comparison with negative control (Figure 2I to K).

### miR-22 promoted mPTP opening induced by TGEV infection

**Figure 3:**
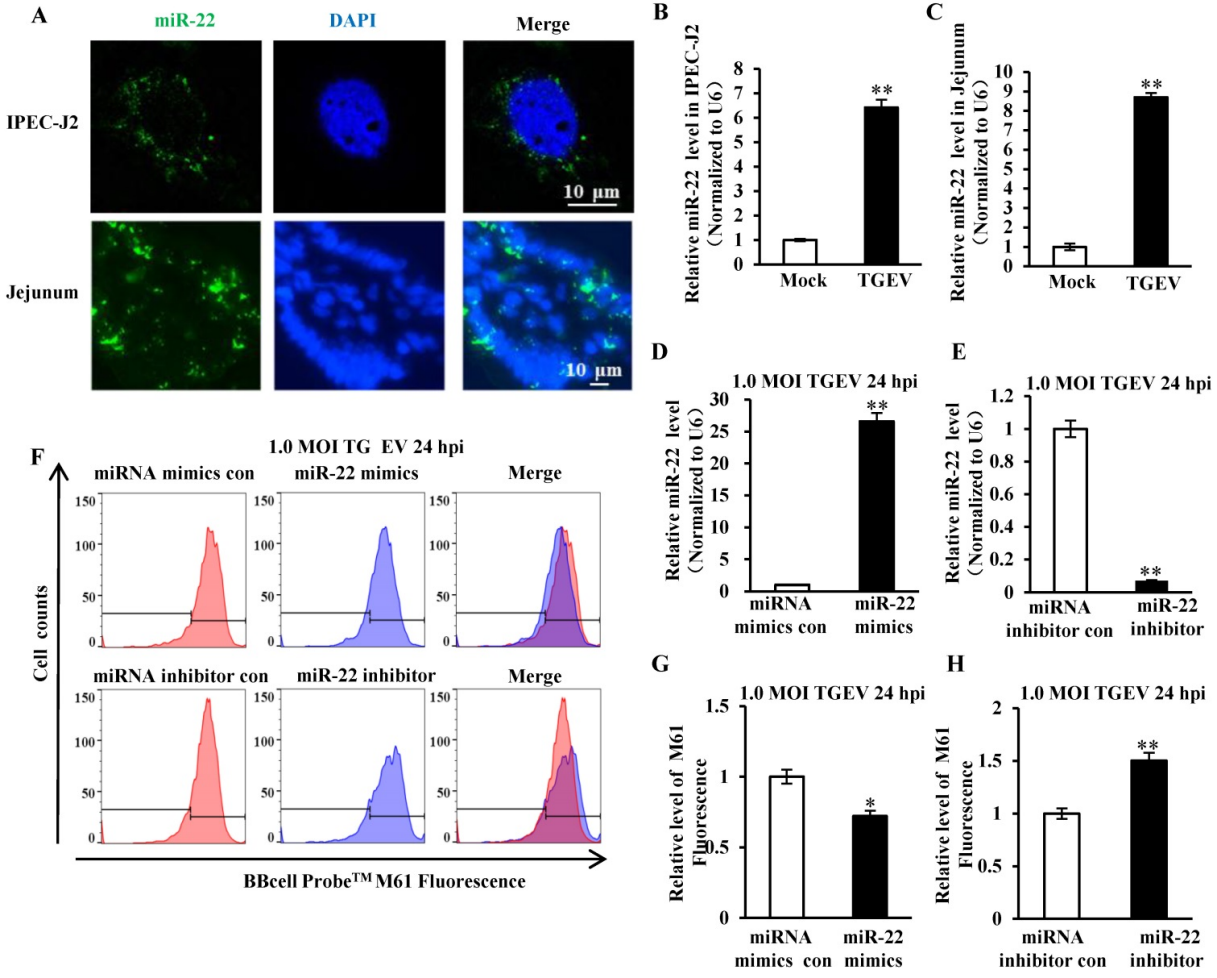
**miR-22 was localized at cytoplasm and promoted mPTP opening.** (A) Localization of miR-22 in IPEC-J2 and jejunum IPEC of piglet. (B) Fold change of miR-22 in IPEC-J2 in response to TGEV infection. (C) Fold change of miR-22 in jejunum IPEC of piglet in response to TGEV infection. (D) Overexpression effect of miR-22 mimics. (E) Silencing effect of miR-22 inhibitor. (F) The effect of miR-22 on TGEV-induced mPTP opening in IPEC-J2. (G) and (H) Quantification of mPTP opening level of Figure 3F. * *p* < 0.05 and ** *p* < 0.01 in comparison with the control.

The secondary structure of pre-miR-22 was predicted using RNAstructure version 6.0.1 and shown in Figure S2A. The FISH analysis showed that miR-22 distributed in cytoplasm of cells *in vitro* and* in vivo* (Figure 3A). Meanwhile, the level of miR-22 in IPEC-J2 and villi of jejunum that were infected with TGEV or Mock infection was evaluated by qRT-PCR and FISH. miR-22 significantly increased both in vitro (Figure 3B and Figure S2B) and in vivo (Figure 3C and Figure S2B) during TGEV infection. To investigate the impact of miR-22 on mPTP opening induced by TGEV infection, IPEC-J2 cells were respectively transfected with miR-22 mimics (or miRNA mimics control, miR-22 inhibitor, miRNA inhibitor control) and subsequently infected with TGEV at 1 MOI for 24 h. The miR-22 level was significantly increased by miR-22 mimics and remarkably decreased by miR-22 inhibitor (Figure 3D and E). The level of mPTP opening was increased by miR-22 mimics and significantly decreased by miR-22 inhibitor (Figure 3F to H).

### circEZH2 suppressed mPTP opening and promoted NF-κB pathway via binding miR-22

**Figure 4:**
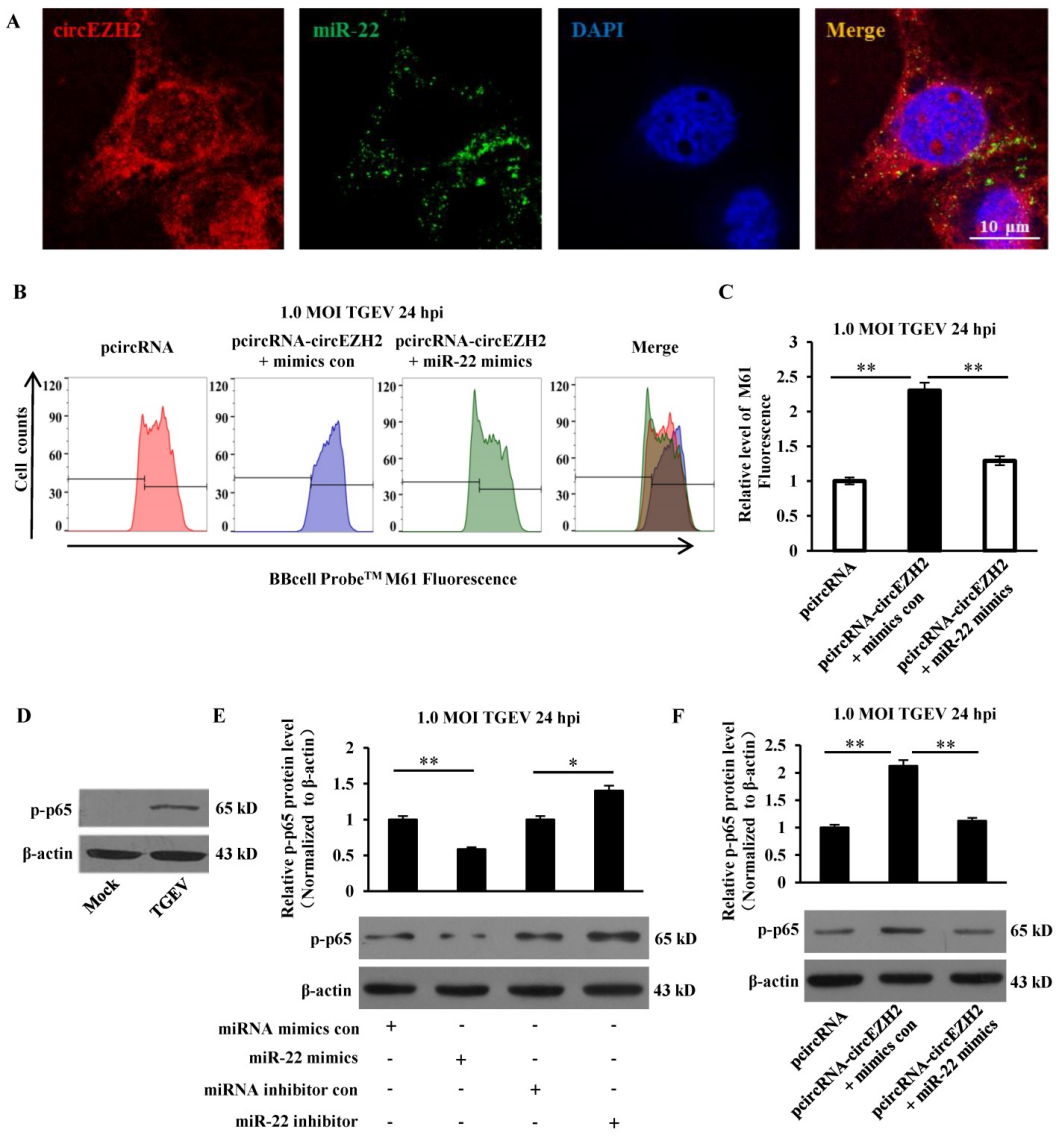
**circEZH2 functions as sponge of miR-22.** (A) Colocalization between miR-22 and circEZH2 in IPEC-J2 infected with TGEV. circEZH2 is shown in red. miR-22 is shown in green. Nucleus is shown in blue. (B) The combined effect of circEZH2 and miR-22 on TGEV-induced mPTP opening in IPEC-J2. (C) Quantification of mPTP opening level of Figure 4B using FACS. (D) The effect of TGEV on p-p65. (E) The effect of miR-22 on p-p65. (F) The combined effect of circEZH2 and miR-22 on p-p65. ** p < 0.01 in comparison with the control.

We previously identified that miR-22 could directly bind to circEZH2 via dual-luciferase reporter assay^21^. To provide further evidence on circEZH2 functions as miR-22 sponge, FISH assay was conducted. Co-localization of circEZH2 and miR-22 was observed in the cytoplasm of IPEC-J2 cells, indicating circEZH2 interacts with miR-22 (Figure 4A). To examine whether circEZH2 suppressed mPTP opening via interacting with miR-22, miR-22 mimics and pcircRNA-circEZH2 were co-transfected into IPEC-J2 cells. The fluorescence intensity was significantly inhibited by miR-22 mimics and pcircRNA-circEZH2 co-transfection compared with pcircRNA-circEZH2 alone (Figure 4B and C). Previous studies have suggested that circEZH2 promoted protein level of p-IκB-α and accumulation of p65 in nucleus in TGEV-infected IPEC-J2 cells^21^. Further analysis showed that TGEV induced accumulation of p-p-65, and miR-22 inhibited this process (Figure 4D and E). The protein level of p-p65 significantly decreased in IPEC-J2 cells co-transfected with miR-22 mimics and pcircRNA-circEZH2 compared with in cells transfected with pcircRNA-circEZH2 alone (Figure 4F). The data suggested that circEZH2 could promote NF-κB pathway and suppress mPTP opening by binding miR-22.

### Analysis of miR-22 targets and mRNAs

**Figure 5:**
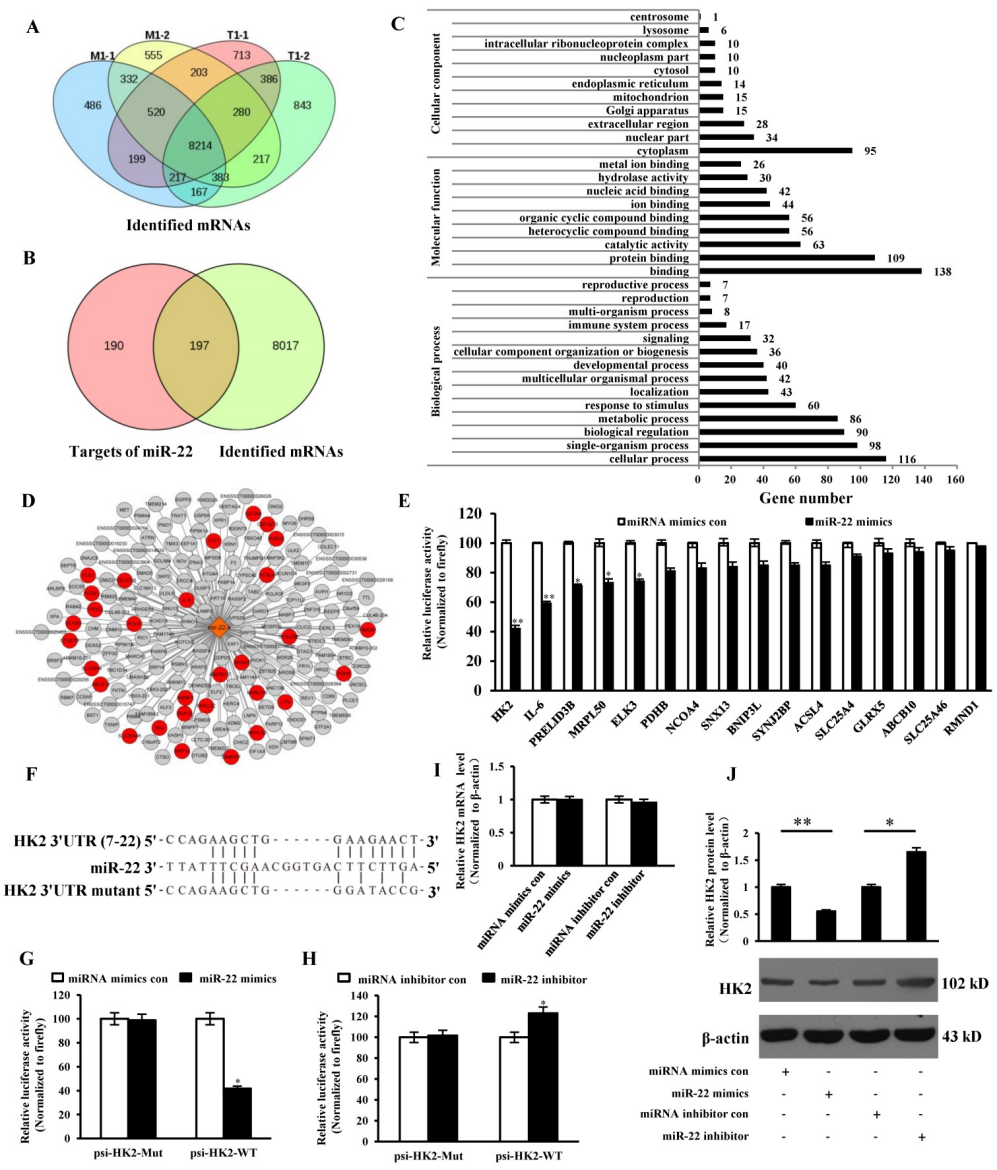
**Correlation analysis between miR-22 target genes and identified mRNAs.** (A) Venn diagram showed the intersection of mRNAs among the four samples. (B) Venn diagram showed the intersection between miR-22 targets and overlapped mRNAs. (C) GO enrichment analysis of miR-22 target genes. (D) The interaction network of miR-22 and its target genes. Red circles indicate target genes of miR-22 localized at mitochondria or participated in the immune system process. (E) The binding ability of miR-22 to 3' UTRs of 16 potential target genes. (F) Schematic overview of mutation of swine HK2 3' UTR sequence. The upper sequence is the binding site of miR-22 in 3' UTR of swine HK2. The middle is the sequence of mature miR-22. The lower sequence is the mutated binding site sequence of miR-22 of HK2 3' UTR. (G) and (H) The relative luciferase activities of psi-HK2-WT and psi-HK2-Mut mediated by miR-22 mimics and miR-22 inhibitor. (I) The relative mRNA level of HK2 in IPEC-J2 treated with miR-22 mimics and miR-22 inhibitor. (J) The effect of miR-22 on expression of HK2. * *p* < 0.05 and ** p < 0.01 in comparison with the control.

The intersection of identified mRNAs of each sample by sequencing was obtained and presented via Venn diagram. 8214 intersective mRNAs were obtained (Table S1 and Figure 5A). 378 target genes of miR-22 were predicted and intersected with the 8214 mRNAs, 197 genes were intersected (Table S2 and Figure 5B). GO enrichment analysis of the 197 genes was performed and presented (Table S3). The result showed that 30 target genes were localized at mitochondria or participated in the immune system process (Figure 5C and D). To identify the targets, 16 target genes of miR-22 that related to mitochondrion and inflammation were selected for identification. The 3' UTR of the 16 target genes were cloned into 3' UTR of dual-luciferase reporter plasmid to gain the recombinant plasmid. The luciferase activities were detected. The results showed that luciferase activity of plasmid containing the 3' UTR of HK2 was markedly lower than that of other targets (Figure 5E). To further identify the direct binding between miR-22 and 3' UTR of HK2 mRNA, the binding site of miR-22 seed sequence at HK2 3' UTR were mutated with the 4-bp substitution (Figure 5F). The constructs and miR-22 mimics (or miRNA mimics control, miR-22 inhibitor, miRNA inhibitor control) were co-transfected into IPEC-J2 cells. The luciferase activities were measured. Compared with the control, introduction of exogenous miR-22 decreased the psi-HK2-WT reporter activity by 45%, while over-expression and inhibition of miR-22 did not affect luciferase activity (Figure 5G and H). The effects of miR-22 on transcription and expression of HK2 were tested using real-time PCR and western blot. The results indicated that the transcriptional level of HK2 was not influenced by miR-22 mimics and inhibitor (Figure 5I). However, the protein level of HK2 was down-regulated by miR-22 mimics and up-regulated by miR-22 inhibitor (Figure 5J). Together, our data conclusively demonstrated that HK2 is a direct target of miR-22 in IPEC-J2 cells.

### si-HK2 promoted mPTP opening induced by TGEV in IPEC-J2

**Figure 6:**
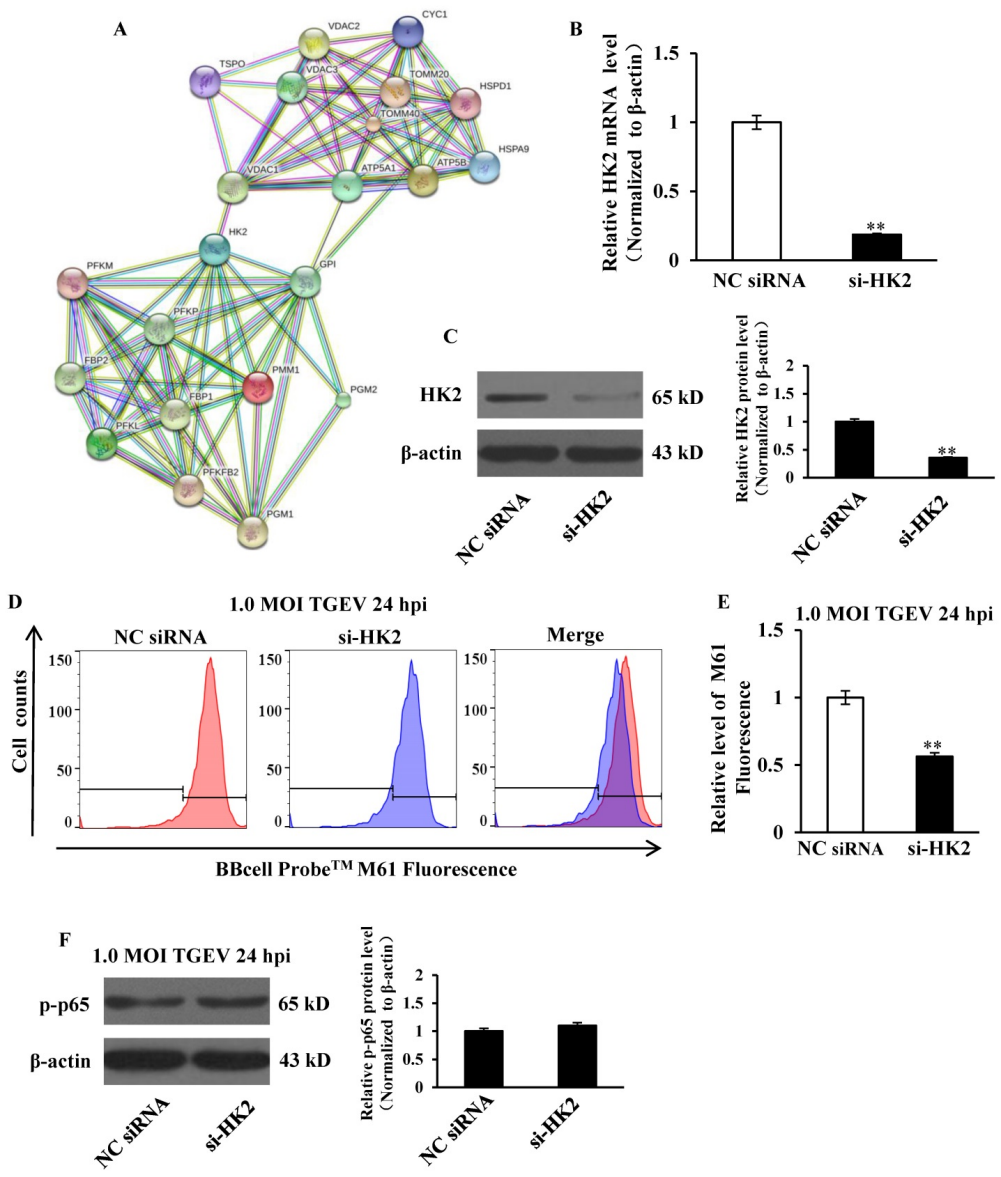
**The effects of HK2 on mPTP opening and NF-κB pathway.** (A) Network node represents protein. Colored nodes represent the query proteins and the first shell of interactors. White nodes represent the second shell of interactions. Small nodes are the proteins with unknown 3D structure. Large nodes are the proteins with known or predicted 3D structure. Lines represent protein-protein interactions. Light blue line represents from curated databases. Purple line represents experimentally determined. Green line represents gene neighborhood. Red line represents gene fusions. Navy blue line represents gene co-occurrence. Yellow represents textmining. Black line represents co-expression. Grey line represents protein homology. (B) and (C) The silencing effect of si-HK2. (D) The effect of si-HK2 on TGEV-induced mPTP opening. (E) Quantification of mPTP opening level of Figure 6D. (F) The effect of siHK2 on p-p65. ** *p* < 0.01 in comparison with the control.

HK2 was introduced into the web-tool STRING to generate protein-protein interaction networks. We found that VDAC1, an important protein in mPTP, might interact with HK2 (Figure 6A). IPEC-J2 cells were transfected with si-HK2 or Negative Control of siRNA (NC siRNA), and subsequently infected with TGEV at 1 MOI for 24 h. The HK2 levels on both mRNA and protein were significantly inhibited by si-HK2 (Figure 6B and C). The fluorescence intensity for BBcellProbe^TM^ M61 was measured using flow cytometry and markedly suppressed by si-HK2 (Figure 6D and E).

### si-HK2 had no effect on NF-κB activation

Previous studies suggested that circEZH2 potentiated TGEV-induced activation of NF-κB pathway through attaching to miR-22. To investigate the effect of HK2 on TGEV-induced NF-κB activation, IPEC-J2 cells were transfected with si-HK2 or Negetive Control (NC siRNA) and subsequently infected with TGEV at 1 MOI for 24 h. The result showed that p-p65 protein level was not affected by siHK2 (Figure 6F).

### miR-22 promoted mPTP opening through suppressed NF-κB pathway and targeting IL-6

**Figure 7:**
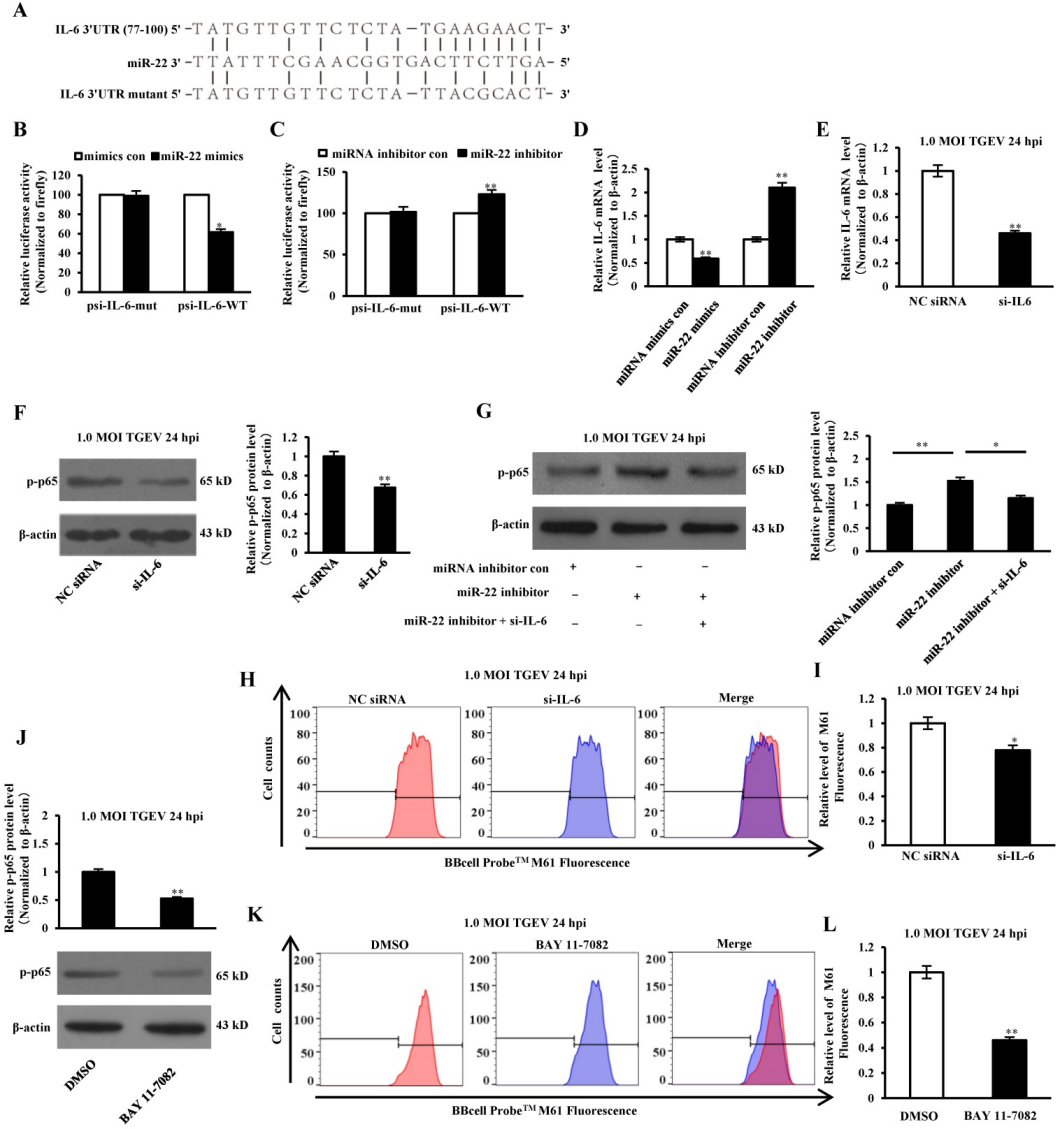
**miR-22 promoted TGEV-induced mPTP opening through suppressing NF-κB pathway and targeting IL-6.** (A) Schematic overview of mutation of swine IL-6 3' UTR sequence. The upper sequence is the binding site of miR-22 in 3' UTR of swine IL-6. The middle is the sequence of mature miR-22. The lower sequence is the mutated sequence of IL-6 3' UTR. (B) and (C) The relative luciferase activities of psi-IL-6-WT and psi-IL-6-Mut mediated by miR-22 mimics and miR-22 inhibitor. (D) The relative mRNA level of IL-6 in IPEC-J2 treated with miR-22 mimics and miR-22 inhibitor. * *p* < 0.05 in comparison with the control. ** *p* < 0.01 in comparison with the control. (E) Silencing effect of si-IL-6. (F) The effect of si-IL-6 on p-p65. (G) The combined effects of miR-22 inhibitor and si-IL-6 on p-p65. (H) The effect of si-IL-6 on TGEV-induced mPTP opening. (I) Quantification of mPTP opening level of Figure 7H. (J) The effect of BAY 11-7082 (NF-κB inhibitor s) on p-p65. (K) The effect of BAY 11-7082 on TGEV-induced mPTP opening. (L) Quantification of mPTP opening level of Figure 7K. * p< 0.05 in comparison with the control. ** *p* < 0.01 in comparison with the control.

IL-6 is a key mediator in response to acute inflammation caused by viral infection, including TGEV, PEDV, HSV, H1N1 influenza virus, and HBV^30^-^33^. Previous studies have confirmed that IL-6 was involved in regulating the NF-κB pathway^34^. To determine the direct interaction between miR-22 and 3' UTR of IL-6 mRNA, the porcine IL-6 3' UTR or its mutant was respectively cloned into the 3' UTR of a Renilla luciferase gene in a luciferase reporter psiCHECK-2 (Figure 7A). The constructs and miR-22 mimics (or miRNA mimics control, miR-22 inhibitor, miRNA inhibitor control) were co-transfected into IPEC-J2 cells. Compared with the control, introduction of exogenous miR-22 decreased the psi-IL-6-WT reporter activity by 62%, while overexpression and knockdown of miR-22 using miR-22 mimics and inhibitor did not affect psi-IL-6-Mut reporter activity (Figure 7B and C). To determine whether miR-22 inhibited IL-6 expression on mRNA level, miR-22 mimics (or miRNA mimics control, miR-22 inhibitor, miRNA inhibitor control) was transfected into IPEC-J2 cells. The mRNA level of IL-6 was assessed by real-time PCR. Introduction of exogenous miR-22 decreased mRNA level of IL-6 in IPEC-J2 cells. Knockdown of endogenous miR-22 using miR-22 inhibitor increased the mRNA level of IL-6 (Figure 7D).To investigate the effect of IL-6 on NF-κB pathway, IPEC-J2 were transfected with si-IL-6 or NC siRNA, and subsequently infected with TGEV at 1 MOI for 24 h. The results showed that p-p65 significantly decreased at protein level by si-IL-6 (Figure 7E and F). To examine whether miR-22 suppressed NF-κB pathway via interacting with IL-6, miR-22 inhibitor and the si-IL-6 were co-transfected into IPEC-J2. The results revealed that p-p65 significantly down-regulated in IPEC-J2 by miR-22 inhibitor and si-IL-6 co-transfection in comparison with miR-22 inhibitor alone (Figure 7G). To investigate the effect of IL-6 on mPTP opening, IPEC-J2 cells were transfected with si-IL-6 or NC siRNA, and subsequently infected with TGEV at 1 MOI for 24 h. The level of mPTP opening was detected using flow cytometry. The results showed that fluorescence intensity for BBcellProbeTM M61 significantly decreased by si-IL-6 (Figure 7H and I), indicating that IL-6 decreased TGEV-induced mPTP opening. IPEC-J2 cells were pretreated with 10 μM NF-κB inhibitor (BAY 11-7082) for 60 min and subsequently infected with TGEV at 1 MOI for 24 h. The level of mPTP opening was detected using flow cytometry. The results showed that fluorescence intensity for BBcellProbeTM M61 significantly decreased by BAY 11-7082 (NF-κB inhibitor) (Figure 7J-L).

## Discussion

IPEC are the target cells for TGEV and contain rich mitochondria contributing to absorption of nutrition and immune response for pathogen infection^35^. TGEV infection causes mitochondrial damage^36^-^38^, which can subsequently result in various pathological processes, including inflammation, cell death. TGEV infection can cause inflammatory response in IPEC-J2 and activate NF-κB pathway 21.

mPTP is the switch of mitochondrial damage and is regulated by NF-κB pathway^27^, ^39^. mPTP is a channel that spans from the outer mitochondrial membrane to the mitochondrial matrix and controls the migration of molecules into and out of mitochondria^40^. Persistent opening of mPTP in response to high concentration of Ca^2+^ can lead to decrease of the mitochondrial membrane potential (△ψm) and makes mitochondria become permeable to molecules less than 1.5 kDa and water 11, 12, 41, 42. Therefore, the opening of mPTP is the key link of mitochondrial damage. Mitochondrial damage can trigger inflammation response 43, 44. We previously reported that circEZH2 activated NF-κB pathway, indicating circEZH2 regulating TGEV-caused inflammation response via NF-κB pathway^21^. It was recently reported that TGEV non-structural protein 2 (nsp2) promotes inflammation via NF-κB pathway^45^. Therefore, TGEV nsp2 may contribute to inflammation via regulating circEZH2. NF-κB pathway is a key pathway that regulates inflammation restrains mitochondrial damage and cell death via suppressing mPTP opening in ventrucular myocytes 27, correlating with our finding that inhibition of NF-κB pathway promoted TGEV-induced mPTP opening.

circRNAs are a class of non-coding RNA and play important roles in many biological and pathological processes, including development, tumorigenesis, viral infection, inflammation via sponging with miRNAs, interacting with proteins, regulating transcription and translation of genes, and regulating parental genes 46, 47. Here, we found that circEZH2 regulated mPTP opening via binding miR-22, while not affecting its parental gene, EZH2. Therefore, it will be of interest to investigate whether circEZH2 play regulatory roles through interacting with proteins or modulating gene transcription or translation.

Voltage-dependent anion channel 1 (VDAC1) is an important regulator of mPTP opening. HK2 interacts with VDAC1 to change the conformation of VDAC1, which leads to an inhibition of mPTP opening 48-50. This correlates with our discovery that silencing HK2, the target of miR-22, aggravates TGEV-induced mPTP opening. However, HK2 does not regulate NK-κB activation. Not only HK2, IL-6 was also identified as the target of miR-22 and suppressed TGEV-induced mPTP opening via activating NF-κB pathway, suggesting that miR-22 regulates TGEV-induced mPTP opening via at least two pathways. Our finding is supported by the discovery that IL-6 reduces hyperoxia-induced mitochondrial damage via Bcl-2-induced Bak-mitofusions interaction and activates NF-κB pathway through STAT3 pathway^34^, ^51^ and provides a new mechanism of miR-22 in mitochondrial function.

In summary, circEZH2 presents a novel function in activating NF-κB and regulating mPTP opening via binding miR-22 and provides a potential novel pharmacologic target in the interventional approach to prevent and control TGEV infection.

## Supplementary Material

Supplementary figures.

Table S1.

Table S2.

Table S3.

Table S4.

## Abbreviations

TGE: transmissible gastroenteritis
TGEV: transmissible gastroenteritis coronavirus
IPEC: porcine intestinal epthelial cell
mPTP: mitochondrial permeability transition pore
NF-κB: nuclear factor kappa-light-chain-enhancer of activated B cells
HK2: Hexokinase 2
IL-6: interleukin 6
CoVs: Coronaviruses
ARDS: acute respiratory distress syndrome
TSCC: tongue squamous cell carcinoma
IRF3: interferon regulating factor 3
HBx: Hepatitis B virus x protein
PEDV: porcine epidemic diarrhea virus
PoRV: Porcine Rotavirus
PCV2: porcine circovirus type 2
PBS: phosphate-buffered saline
HE: hematoxylin-eosin
HRP: Horseradish peroxidase
DMEM: Dulbecco's Minimal Essential Medium
PVDF: polyvinylidene difluoride
ECL: enhanced chemiluminescence
MOI: multiplicity of infection
DAPI: 4,6-diamidino-2-phenylindole
PMSF: phenylmethyl sulonylfluoride
FISH: Fluorescence in situ hybridization
FACS: Fluorescence Activated Cell Sorting
