# Supplementary material for: Circular RNA CircEZH2 Suppresses Transmissible Gastroenteritis Coronavirus-induced Opening of Mitochondrial Permeability Transition Pore via Targeting MiR-22 in IPEC-J2
Source: Int J Biol Sci. 2019 Jul 25;15(10):2051–64. doi: 10.7150/ijbs.36532 (PMC6775298; doi:10.7150/ijbs.36532)
Supplement: Supplementary file 1 — Supplementary figures. [file ijbsv15p2051s1.pdf]

Supplementary Information

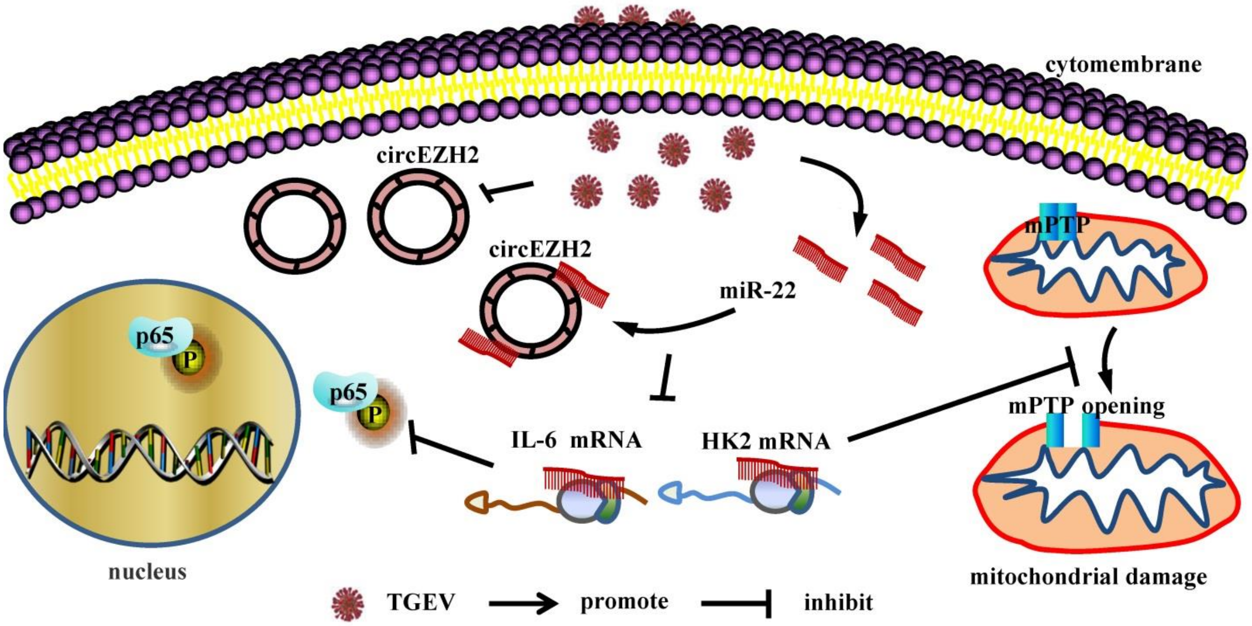

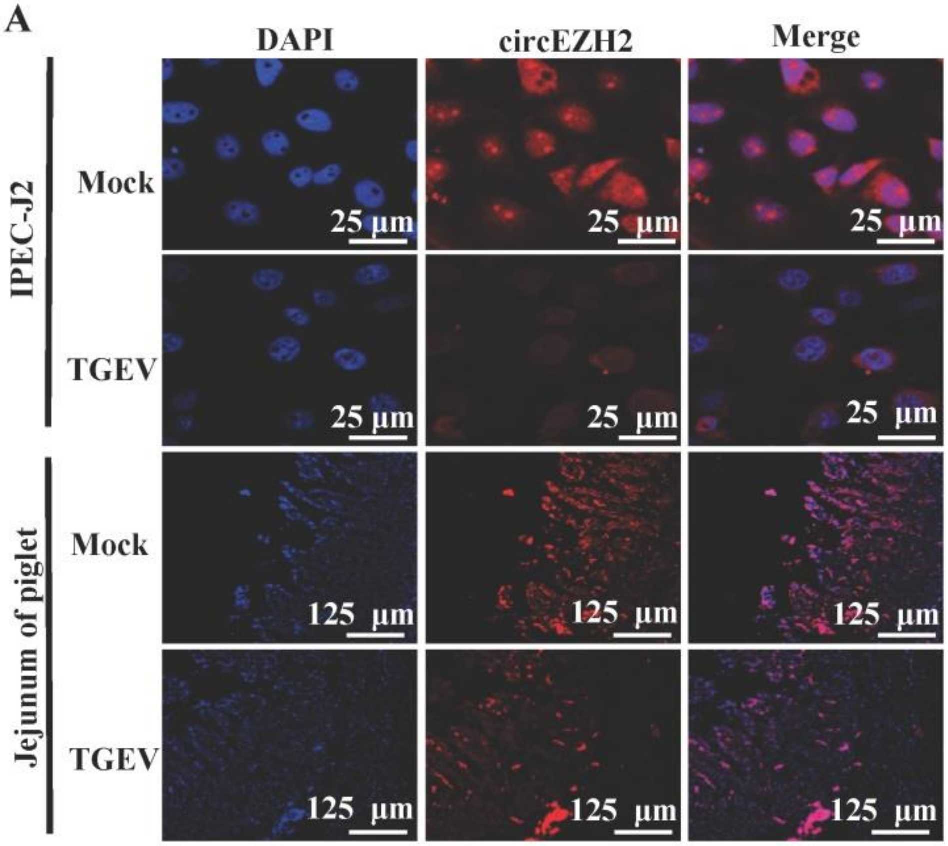

Figure S1 circEZH2 level is down-regulated in response to TGEV infection *in vivo* and *in vitro* analyzed by FISH.

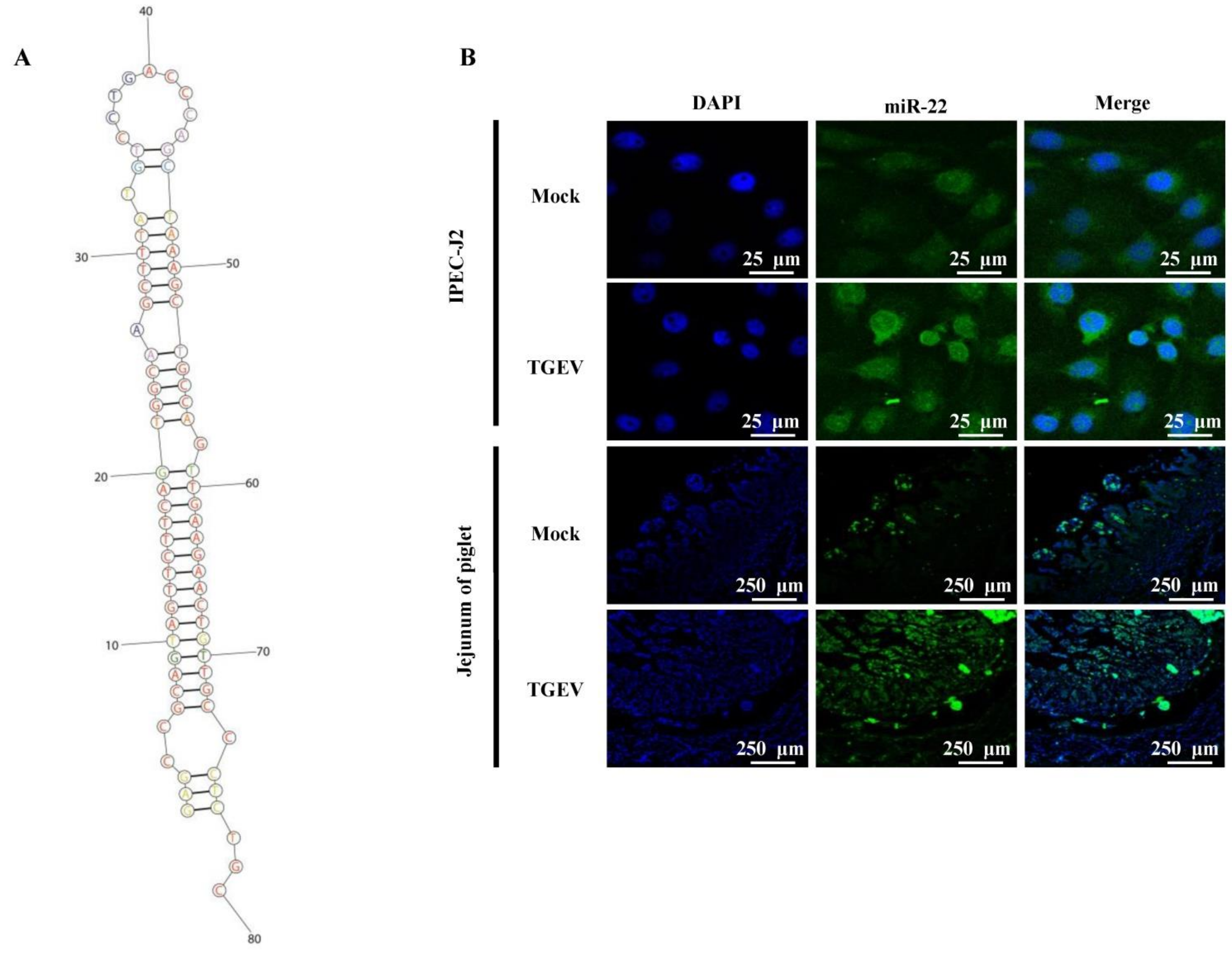

Figure S2 miR-22 level is up-regulated response to TGEV infection *in vivo* and *in vitro* analyzed by FISH.

(A) The secondary structure of pre-miR-22. (B) miR-22 level increased in response to TGEV infection..

Table S1 The overlapped mRNAs in four samples.

Table S2 The intersection between miR-22 target genes and mRNAs.

Table S3 Sequences for probes, miRNA mimics and inhibitors, siRNAs.

Table S4 Sequences for qRT-PCR and PCR primers.
